# Distinct Responses of Rare and Abundant Microbial Taxa to *In Situ* Chemical Stabilization of Cadmium-Contaminated Soil

**DOI:** 10.1128/mSystems.01040-21

**Published:** 2021-10-12

**Authors:** Min Xu, Qiaoyun Huang, Zhenqian Xiong, Hao Liao, Zhenguang Lv, Wenli Chen, Xuesong Luo, Xiuli Hao

**Affiliations:** a State Key Laboratory of Agricultural Microbiology, Huazhong Agricultural University, Wuhan, China; b Hubei Key Laboratory of Soil Environment and Pollution Remediation, Huazhong Agricultural University, Wuhan, China; c State Environmental Protection Key Laboratory of Soil Health and Green Remediation, Wuhan, China; University of Waterloo

**Keywords:** cadmium-contaminated soil, community assembly, ecosystem multifunctionality, microbial rare and abundant taxa, stabilizing amendments

## Abstract

Soil microorganisms, which intricately link to ecosystem functions, are pivotal for the ecological restoration of heavy metal-contaminated soil. Despite the importance of rare and abundant microbial taxa in maintaining soil ecological function, the taxonomic and functional changes in rare and abundant communities during *in situ* chemical stabilization of cadmium (Cd)-contaminated soil and their contributions to the restoration of ecosystem functions remain elusive. Here, a 3-year field experiment was conducted to assess the effects of five soil amendments (CaCO_3_ as well as biochar and rice straw, individually or in combination with CaCO_3_) on rare and abundant microbial communities. The rare bacterial community exhibited a narrower niche breadth to soil pH and Cd speciation than the abundant community and was more sensitive to environmental changes altered by different soil amendments. However, soil amendments had comparable impacts on rare and abundant fungal communities. The assemblies of rare and abundant bacterial communities were dominated by variable selection and stochastic processes (dispersal limitation and undominated processes), respectively, while assemblies of both rare and abundant fungal communities were governed by dispersal limitation. Changes in soil pH, Cd speciation, and soil organic matter (SOM) by soil amendments may play essential roles in community assembly of rare bacterial taxa. Furthermore, the restored ecosystem multifunctionality by different amendments was closely related to the recovery of specific keystone species, especially rare bacterial taxa (*Gemmatimonadaceae* and *Haliangiaceae*) and rare fungal taxa (*Ascomycota*). Together, our results highlight the distinct responses of rare and abundant microbial taxa to soil amendments and their linkage with ecosystem multifunctionality.

**IMPORTANCE** Understanding the ecological roles of rare and abundant species in the restoration of soil ecosystem functions is crucial to remediation of heavy metal-polluted soil. Our study assessed the efficiencies of five commonly used soil amendments on recovery of ecosystem multifunctionality and emphasized the relative contributions of rare and abundant microbial communities to ecosystem multifunctionality. We found great discrepancies in community composition, assembly, niche breadth, and environmental responses between rare and abundant communities during *in situ* chemical stabilization of Cd-contaminated soil. Application of different soil amendments triggered recovery of specific key microbial species, which were highly related to ecosystem multifunctionality. Together, our results highlighted the importance of rare bacterial as well as rare and abundant fungal communities underpinning restoration of soil ecosystem multifunctionality during the Cd stabilization process.

## INTRODUCTION

Intense anthropogenic activities and rapid industrialization accelerate heavy metal pollution in agricultural soil, leading to a great threat to global food security, ecosystem, and human health. Cadmium (Cd), in particular, a nonessential toxic metal that ranks 7th among 20 strong toxins, is one of the most concerned priority pollutants due to its high risk of human exposure and long residence time in soil (1). At present, the widespread occurrence of Cd contamination in agricultural soils has been reported in many regions of the world, including Thailand, India, China, and Japan (2). In China, approximately 1.3 × 10^5^ ha of farmlands is contaminated by Cd, accounting for 20% of the total farmland area (3).

With increasing calls for restoration of Cd-contaminated agricultural soil, research efforts have been made to find sustainable and effective remedial solutions over the past few decades (2–4). Compared to physical and biological remediation strategies (e.g., soil mixing, electrokinetic, phytoremediation, and microbial remediation), *in situ* chemical stabilization has been widely used in the remediation of Cd-contaminated soils due to its efficiency and low-cost in decreasing Cd toxicity and bioavailability (5). The choice and application strategies of Cd-stabilizing agents are of particular importance for Cd stabilization efficiency *in situ* since their properties and underlying stabilizing mechanisms vary greatly. Organic amendments (such as biochar, compost, and straw) stabilize Cd and other metals in soil via forming stable organic ligand-metal complexes (4). Liming materials (such as limestone and calcium hydroxide) can effectively stabilize most metals in soil by increasing soil pH and negatively charged sorption sites of soil colloid and organic matter (6). The application of clay materials (such as sepiolite and zeolite) to Cd stabilization is mainly based on their high surface areas and excellent ion exchange capacities (7). Among various Cd stabilizing agents, limestone (primarily CaCO_3_), biochar, and crop straw are highly recommended in previous studies due to their multiple effects on soil restoration, including reducing Cd bioavailability, alleviation of soil acidification, and enhancing soil ecological functions (8, 9). To achieve a better performance, combinations of different amendments are also recommended (10).

Diverse microorganisms in soil play critical roles in maintaining multiple ecosystem functions simultaneously (“ecosystem multifunctionality” hereafter), including nutrients cycling, organic matter decomposition, soil health, and crop productivity (11). In natural environments, the abundance and distribution of species in microbial communities is uneven, with a few abundant species and a large number of rare species (12). Traditional studies mainly focus on the abundant members of microbial communities due to their contributions to biomass and nutrient cycling in ecosystems (13, 14). However, recent studies have emphasized the ecological importance of rare taxa in maintaining microbial diversity and ecosystem function (15, 16). As part of the microbial “seed bank,” rare species exhibit high diversity and functional redundancy and, thus, serve as functional insurance in microbial community (17). Both abundant and rare species interact intensively, either intra or interkingdom and constitute complex ecological networks. Some species, regardless of their abundance, occupy key positions (e.g., hubs and connectors) in the ecological networks and are considered as keystone species essential for the stability of community structure (18). Recently, network analysis-based approaches have been used to infer the potential interactions, identify keystone taxa, and decipher the relationship between ecological clustering and environmental factors in many ecosystems (19–21). The keystone species have been shown to be closely pertinent to attributes or functional genes involved in multiple ecological processes, including nutrient cycling, carbon turnover, and crop productivity (19, 22). In particular, the rare taxa may function as keystone species responsible for the maintenance of community structure and ecosystem multifunctionality (23).

The responses of abundant and rare species to environmental disturbances are not always consistent (24, 25). Abundant species normally occupy a wider niche breadth and can utilize more types of resources, which enable them to be more adaptive to environmental changes than rare species (26). For instance, due to the discrepancy in resistance to heavy metals, nearly all rare taxa in pristine soil were eliminated by heavy metal pollution, leading to a severe reduction of bacterial diversity (27). However, contradictory results were also reported in other studies showing that the diversity and community composition of rare taxa are more stable when suffering climate change (25) and other disturbances, such as copper stress, heat shock, freezing-thawing, and mechanical disturbance (28). These unaffected rare taxa might be dormant or extremely slow growing but could be activated or become dominant when the environment is favorable (28, 29). In addition, distinct assembly processes of abundant and rare communities have been found in many ecosystems, likely due to their differential responses to environmental changes (30, 31). During *in situ* chemical stabilization process, applications of stabilizing amendments lead to multiple changes in soil properties, including metal speciation, soil pH, and available nutrients (32). These changes may consequently alter the assembly and distribution patterns of abundant and rare species in the microbial community, leading to unknown outcomes for ecosystem multifunctionality. Given that the abundant and rare species may differentially affect functional attributes, distinguishing the roles of abundant and rare taxa in restoration of ecosystem multifunctionality in Cd-contaminated soil is of importance but remains largely unexplored.

We hypothesize that the rare community could be more sensitive to amendment-induced changes in Cd bioavailability and soil properties than the abundant community, and the recovery of rare taxa may play vital roles in restoration of soil ecosystem multifunctionality. To test our hypothesis, we conducted a 3-year field experiment applied with five soil amendments (CaCO_3_ as well as biochar and rice straw, individually or in combination with CaCO_3_). The impacts of amendments on composition shifts, niche breadth, and assembly processes of microbial abundant and rare communities were characterized to uncover microbial responses and the mechanisms underlying amendment-induced effects on ecosystem multifunctionality. In particular, we aimed to (i) compare the responses of abundant and rare taxa of bacterial and fungal communities to different soil amendments, (ii) evaluate their contributions to soil ecosystem multifunctionality, and (iii) identify keystone species of abundant and rare communities, which are associated with soil ecosystem multifunctionality in different stabilizing treatments.

## RESULTS

**FIG 1:**
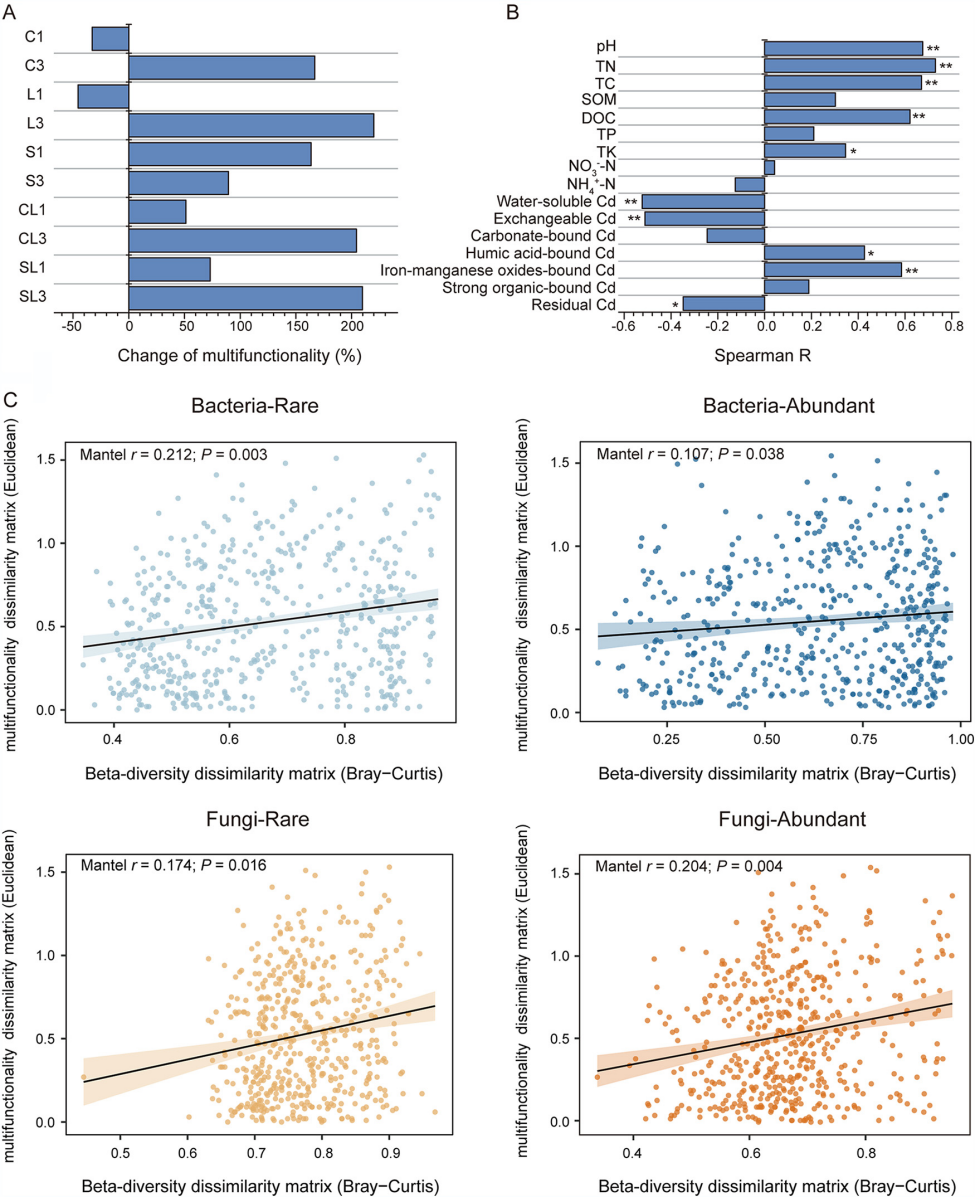
Ecosystem multifunctionality and its influencing factors. (A) The fold change of ecosystem multifunctionality in different treatments relative to the control. (B) Spearman correlation between ecosystem multifunctionality and edaphic factors. (C) Mental correlation between community dissimilarity and ecosystem multifunctionality. Significant correlations were labeled as follows: **, *P* < 0.01; *, *P* < 0.05. Treatments include single application of biochar (C1), CaCO_3_ (L1), straw (S1), CaCO_3_ together with biochar (CL1) and CaCO_3_ together with straw (SL1), and triple application of biochar (C3), CaCO_3_ (L3), straw (S3), CaCO_3_ together with biochar (CL3), and CaCO_3_ together with straw (SL3).

Our result showed that the application of most soil amendments enhanced ecosystem multifunctionality, and, among them, triple application of CaCO_3_ and CaCO_3_ together with biochar/straw exhibited the greatest positive impacts (Fig. 1A). Spearman correlation analysis indicated that ecosystem multifunctionality was positively correlated with soil pH, total carbon (TC), total nitrogen (TN), dissolved organic carbon (DOC), total potassium (TK), humic acid-bound Cd, and Fe-Mn oxide-bound Cd (*P* < 0.05) but had a negative correlation with two labile fractions of Cd (water-soluble and exchangeable) and residual Cd (*P* < 0.05; Fig. 1B). Despite the weak correlations, the Mantel test showed that the compositional changes of rare bacterial and abundant fungal communities were more correlated with ecosystem multifunctionality compared to abundant bacterial and rare fungal communities (*P* < 0.01; Fig. 1C). This result indicated that changes in microbial rare and abundant communities may have differential impacts on soil ecosystem multifunctionality. To further understand the roles of microbial rare and abundant communities, we explored their responses to different amendments and the linkage with soil ecosystem multifunctionality shown below.

### Responses of microbial rare and abundant taxa to soil amendments.

**FIG 2:**
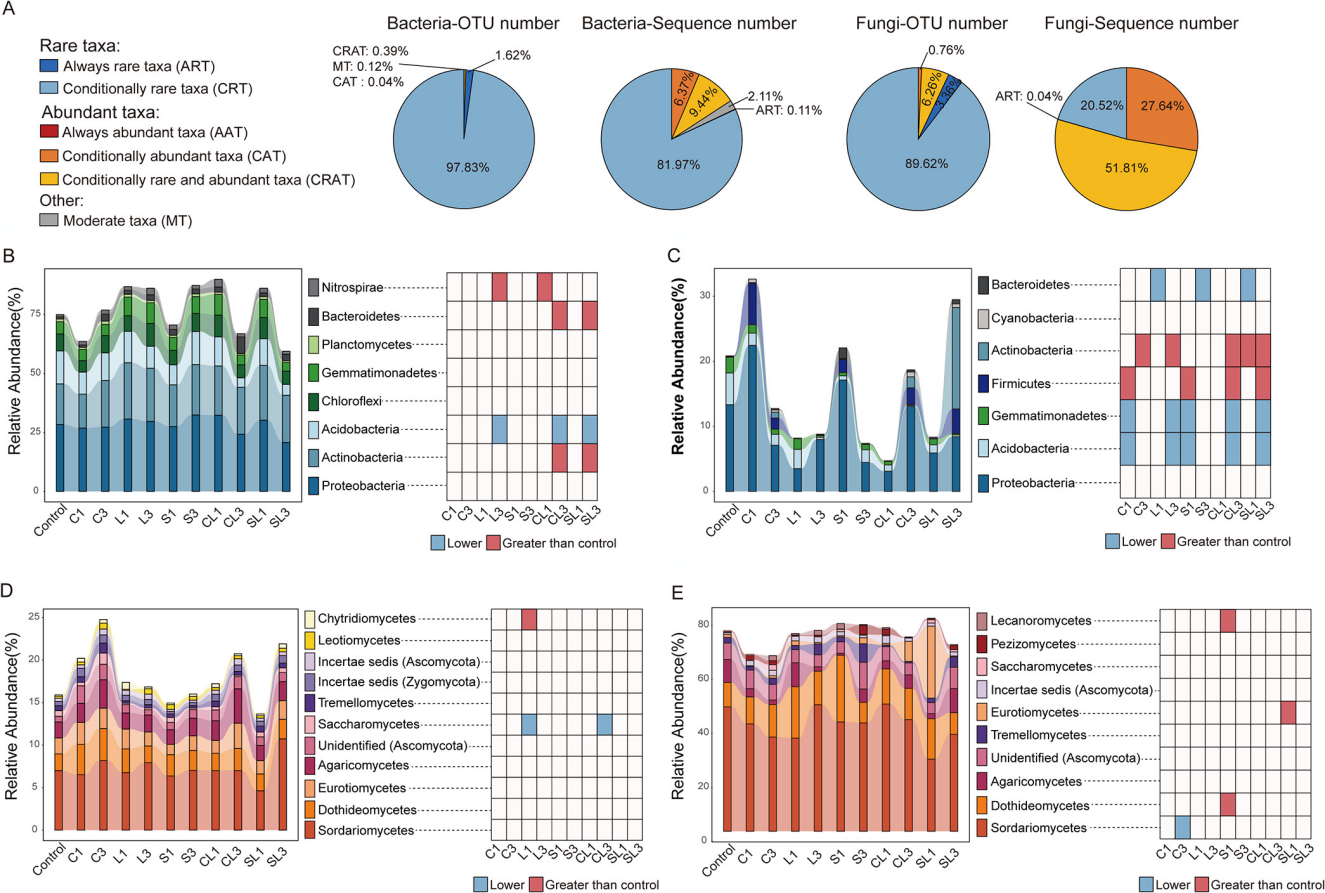
Changes in microbial community composition of rare and abundant taxa by soil amendments. (A) Proportion of six categories of microbial taxa to the overall OTU and sequence numbers. Relative abundance of rare (B) and abundant (C) bacterial phyla in different treatments. Only bacterial phyla with the relative abundance of >0.5% were shown in the figure. Relative abundance of classes (>0.1% abundance) in rare (D) and abundant (E) fungal communities. Bacterial phyla or fungal classes that were significantly different in relative abundances between treatment and control were identified using DeSeq (α = 0.05) and illustrated in heatmaps. Phylum/class with significantly higher or lower abundance in treatment than control was colored in red or blue, respectively. Treatments include single application of biochar (C1), CaCO_3_ (L1), straw (S1), CaCO_3_ together with biochar (CL1), and CaCO_3_ together with straw (SL1) and triple application of biochar (C3), CaCO_3_ (L3), straw (S3), CaCO_3_ together with biochar (CL3), and CaCO_3_ together with straw (SL3).

Rare bacterial taxa accounted for the majority of total operational taxonomic units (OTUs) (99.5%) and sequences (82.1%), while abundant bacterial taxa only comprised 0.4% of the total OTUs and 15.8% of the total sequences (Fig. 2A). In contrast to bacteria, the fungal community was dominated by abundant taxa. Although only less than 7.0% of all OTUs were classified as abundant fungal taxa, these taxa accounted for 79.45% of total sequences (Fig. 2A).

Among 35 rare bacterial phyla, *Proteobacteria*, *Actinobacteria*, and *Acidobacteria* were dominant, accounting for 58.5% of total sequences (Fig. 2B). *Proteobacteria* were also dominant in the abundant community, accounting for 9.7% of total sequences (Fig. 2C). The relative abundances of most rare phyla were quite stable, and only several rare phyla were affected by soil amendments. For instance, rare *Nitrospirae* were enhanced by triple application of CaCO_3_ and single application of CaCO_3_ with biochar, while *Bacteroidetes* and *Actinobacteria* showed significantly higher relative abundances in triple application of CaCO_3_ together with biochar/straw treatments than those in the control (Fig. 2B). Conversely, abundant bacterial phyla were more susceptible to soil amendments than the rare ones, with dramatic changes in the relative abundances of abundant *Actinobacteria*, *Firmicutes*, *Gemmatimonadetes*, and *Acidobacteria* in different amendments (Fig. 2C). Yet, consistent changes were observed for the abundant phyla in single application of biochar and straw treatments, with a significant increase in *Firmicutes* but a decrease in *Gemmatimonadetes* and *Acidobacteria*. Similar results were also obtained in triple application of CaCO_3_ with biochar/straw treatments but with additional enhanced effects on *Actinobacteria* (Fig. 2C). In addition, the mean values of standardized effect size measure of the mean nearest taxon distance (SES.MNTD) were significantly lower in the rare bacterial community than in the abundant community, indicating a more closely phylogenetic clustering of the rare bacterial community (see Fig. S1A in the supplemental material; *P* < 0.001)

10.1128/mSystems.01040-21.1 FIG S1 Boxplot showing differences in the SES.MNTD values between rare and abundant communities in bacteria (A) and fungi (B). Download FIG S1, TIF file, 0.5 MB. Copyright © 2021 Xu et al. 2021 Xu et al. https://creativecommons.org/licenses/by/4.0/ This content is distributed under the terms of the Creative Commons Attribution 4.0 International license.

Compared to bacteria, the responses of rare and abundant fungal taxa to soil amendments were stable. The rare fungal community was dominated by class *Sordariomycetes* (7.3%) and *Dothideomycetes* (2.5%) (Fig. 2D), which was similar to the abundant community, with *Sordariomycetes* and *Dothideomycetes* accounting for 51.3% of total sequences (Fig. 2E). We observed a significantly lower abundance of rare *Saccharomycetes* in single application of CaCO_3_ and triple application of CaCO_3_ with biochar treatments but a higher abundance of rare *Chytridiomycetes* in single application of CaCO_3_ compared to that of the control (Fig. 2D). For abundant fungal taxa, a single application of straw enhanced the relative abundances of *Lecanoromycetes* and *Dothideomycetes*. *Eurotiomycetes* also showed an enhanced relative abundance in single application of CaCO_3_ together with straw treatment. Only *Sordariomycetes* exhibited a significantly lower relative abundance in triple application of biochar treatment than the control (*P* < 0.05; Fig. 2E). Similar to the bacterial community, the mean values of SES.MNTD in the rare fungal community were significantly lower than those in the abundant community (Fig. S1B; *P* < 0.05).

### Environmental responses of microbial rare and abundant communities.

**FIG 3:**
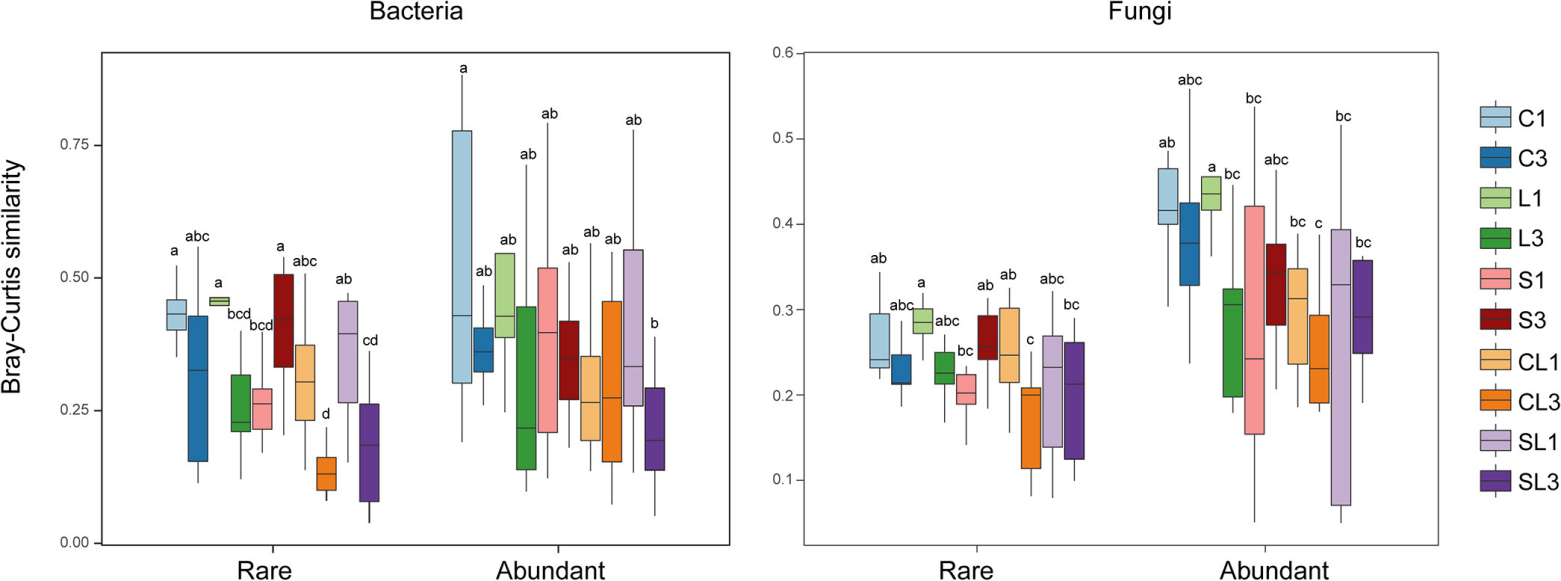
Comparison of rare and abundant community similarities between different treatments. The pairwise similarity between communities in each treatment and the control was calculated based on the Bray-Curtis metric. A greater value indicates higher similarity. Different letters represent significant differences of Bray-Curtis similarity between treatments (*P* < 0.05). Treatments include single application of biochar (C1), CaCO_3_ (L1), straw (S1), CaCO_3_ together with biochar (CL1), and CaCO_3_ together with straw (SL1) and triple application of biochar (C3), CaCO_3_ (L3), straw (S3), CaCO_3_ together with biochar (CL3), and CaCO_3_ together with straw (SL3).

Application of most soil amendments did not alter the α-diversity of rare and abundant bacterial and fungal communities. Only single application of CaCO_3_ with biochar and triple application of straw treatments significantly decreased the Shannon index of the abundant bacterial community (*P* < 0.05) (see Table S1 in the supplemental material). However, soil amendments had a stronger influence on community similarity than α-diversity, especially on the rare bacterial community (Fig. 3). The lower similarity of both the rare bacterial and fungal communities than that of the corresponding abundant communities indicated that β-diversity of rare taxa was more susceptible to soil amendments (Fig. 3). Further, different soil amendments showed distinct impacts on community similarity of rare and abundant taxa. For instance, triple application of CaCO_3_ together with straw had the greatest impact on community similarity of abundant bacterial taxa, while the impacts of a single application of straw, triple application of CaCO_3_, and triple application of CaCO_3_ together with biochar/straw on the similarity of the rare bacterial community were stronger. In contrast to bacteria, the similarities of both rare and abundant fungal communities varied largely with soil amendments. The highest similarity for both rare and abundant fungal communities was observed in single application of CaCO_3_ treatment.

10.1128/mSystems.01040-21.5 TABLE S1 Summary of α-diversity index of microbial rare and abundant communities. Download Table S1, DOCX file, 0.02 MB. Copyright © 2021 Xu et al. 2021 Xu et al. https://creativecommons.org/licenses/by/4.0/ This content is distributed under the terms of the Creative Commons Attribution 4.0 International license.

To further identify the edaphic factors affecting rare and abundant communities, we carried out a Monte Carlo permutation test. Both rare and abundant communities of bacteria and fungi were found to be significantly affected by soil pH, TN, TC, and exchangeable Cd (Table 1; *P* < 0.05). Not surprisingly, soil pH was the most important attribute affecting bacterial and fungal communities. Soil organic matter (SOM) showed significant correlations with rare and abundant bacterial communities, while DOC was only significantly correlated with the abundant fungal community (*P* < 0.05). Both rare and abundant bacterial communities, as well as the abundant fungal community, were significantly correlated with Cd speciation (Table 1; *P* < 0.05). Less correlation between Cd speciation and rare fungal community suggested that rare fungal taxa may be more resistant to Cd stress. This result was further confirmed by a broader niche breadth of rare fungal taxa to Cd speciation than the abundant one (see Fig. S2 in the supplemental material).

**TABLE 1:** Impacts of edaphic factors on rare and abundant communities (Monte Carlo permutation test)

Factor	Bacteria				Fungi			
	Rare		Abundant		Rare		Abundant	
	*R* ^2^	*P*	*R* ^2^	*P*	*R* ^2^	*P*	*R* ^2^	*P*
pH	0.700	0.001	0.530	0.001	0.526	0.001	0.675	0.001
TN	0.234	0.020	0.256	0.006	0.432	0.001	0.347	0.001
TC	0.277	0.006	0.226	0.021	0.298	0.017	0.206	0.031
SOM	0.204	0.017	0.415	0.001	0.011	0.741	0.089	0.184
DOC	0.109	0.206	0.140	0.101	0.079	0.256	0.522	0.001
TP	0.015	0.808	0.040	0.538	0.081	0.279	0.069	0.358
TK	0.070	0.346	0.023	0.716	0.206	0.028	0.071	0.338
NO_3_^−^-N	0.034	0.580	0.049	0.492	0.001	0.994	0.026	0.705
NH_4_^+^-N	0.240	0.011	0.134	0.112	0.127	0.138	0.004	0.958
Water-soluble Cd	0.473	0.001	0.373	0.002	0.118	0.157	0.322	0.008
Exchangeable Cd	0.350	0.001	0.249	0.023	0.350	0.003	0.359	0.002
Carbonate-bound Cd	0.210	0.023	0.297	0.013	0.160	0.099	0.261	0.042
Humic acid-bound Cd	0.514	0.001	0.309	0.008	0.163	0.083	0.359	0.002
Fe-Mn oxide-bound Cd	0.506	0.001	0.285	0.010	0.320	0.003	0.454	0.001
Strong organic-bound Cd	0.028	0.668	0.130	0.136	0.109	0.142	0.156	0.088
Residual Cd	0.103	0.209	0.069	0.348	0.180	0.040	0.100	0.215

10.1128/mSystems.01040-21.2 FIG S2 Environmental breadth estimated by the threshold values of rare and abundant taxa in response to environmental variables. Download FIG S2, TIF file, 0.8 MB. Copyright © 2021 Xu et al. 2021 Xu et al. https://creativecommons.org/licenses/by/4.0/ This content is distributed under the terms of the Creative Commons Attribution 4.0 International license.

### Assembly processes of microbial rare and abundant communities.

**FIG 4:**
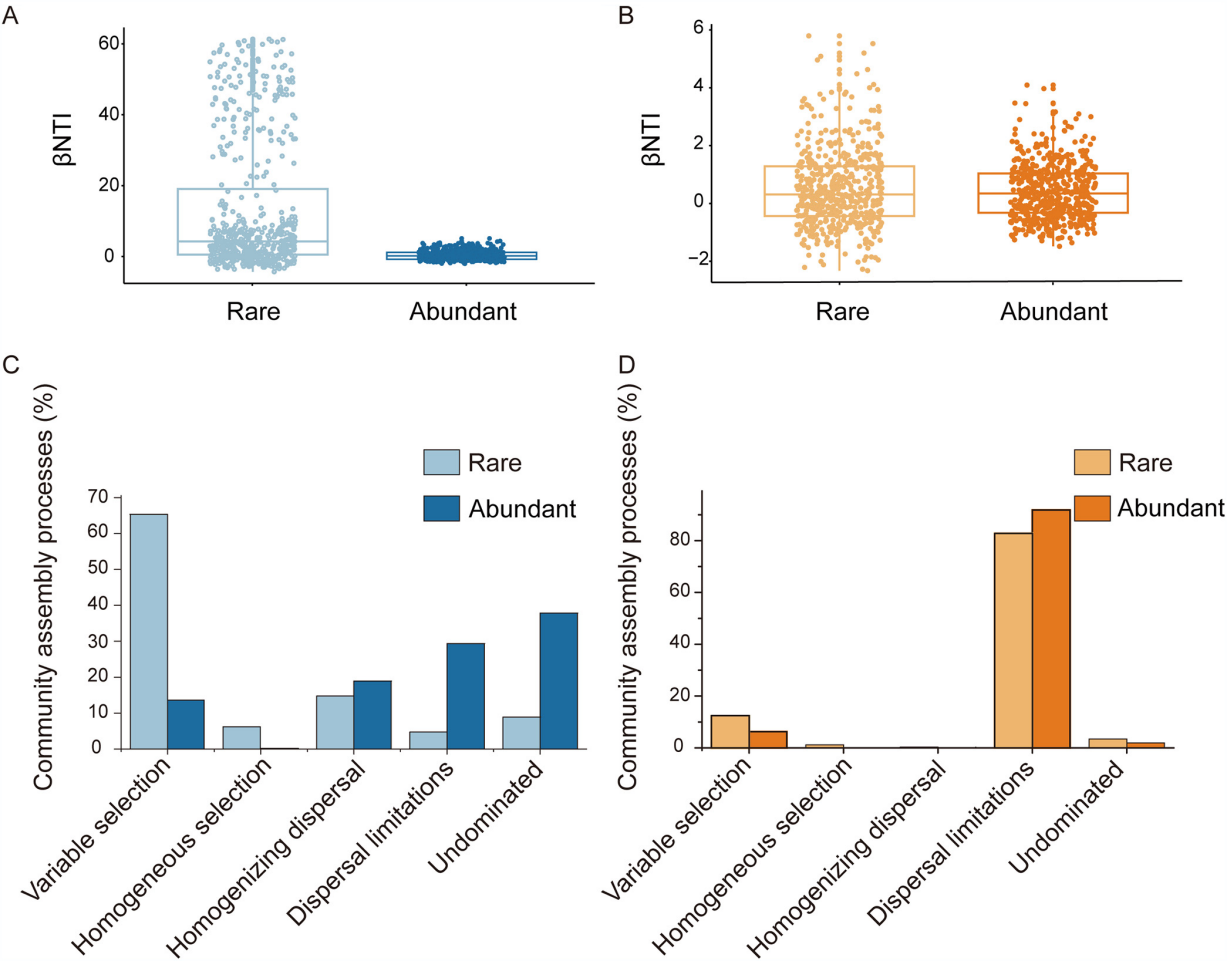
Assembly processes of microbial rare and abundant communities. Boxplot showing βNTI patterns of all pairwise bacterial (A) and fungal (B) communities between different treatments. The proportion of different assembly processes in rare and abundant communities of bacteria (C) and fungi (D).

The β-mean nearest taxon index (βNTI) was used to assess the potential roles of deterministic and stochastic processes in shaping bacterial and fungal community assembly during the stabilization process. In the rare bacterial community, the βNTI value varied from −4.3 to 61.3, with only 30.8% of βNTIs being between −2 and 2, indicating a largely deterministic assembly (Fig. 4A). By contrast, the βNTI value of abundant bacterial community for all possible pairwise varied from −2.0 to 5.1, with 86.2% of βNTI values between −2 and 2, suggesting that the stochastic process was dominant in the assembly of abundant bacterial community (Fig. 4A). For fungal community, the βNTI values of both rare and abundant communities varied from −2.3 to 5.8 and −1.5 to 4.1, with 86.6% and 93.9% of βNTIs between −2 and 2, respectively (Fig. 4B). These observations indicated that the assembly of rare and abundant fungal communities was dominated by stochastic processes. We further quantified the relative contributions of major ecological processes governing the assembly of the rare and abundant communities. For the bacterial community, variable selection (65.34%) was pronounced in the rare community, while the abundant community was more affected by undominated processes (37.88%) and dispersal limitation (29.36%) (Fig. 4C). In contrast, both rare and abundant fungal communities were dominated by dispersal limitation (82.77% and 91.86%, respectively) (Fig. 4D).

As variable selection dominated the assembly of the rare bacterial community, a Mantel test was conducted to explore the major limited environmental factors for assembly processes. Soil pH, TC, SOM, humic acid-bound Cd, and Fe-Mn oxide-bound Cd were found to be significant predictors for assembly processes of the rare bacterial community (see Table S2 in the supplemental material; *P* < 0.05). Except for humic acid-bound Cd, these predictors showed significant and positive correlations with pairwise comparisons of βNTI values for the rare bacterial community (see Fig. S3 in the supplemental material; *P* < 0.05), indicating that the relative influence of deterministic assembly in the rare bacterial community increased with the increase in divergence of these edaphic factors. It is worth noting that only βNTI of the rare bacterial community had significant correlation with soil pH and humic acid-bound or Fe-Mn oxide-bound Cd, suggesting that soil pH and Cd stress would affect the assembly process of the rare bacterial community rather than the abundant community (Table S2).

10.1128/mSystems.01040-21.3 FIG S3 Relationships between β-nearest taxon index (βNTI) and differences in edaphic factors for the rare bacterial community. Linear regressions models (shown as black lines) and associated correlation coefficients are provided in each panel. Horizontal-dashed lines indicate the βNTI significance thresholds of +2 and −2. Download FIG S3, TIF file, 1.7 MB. Copyright © 2021 Xu et al. 2021 Xu et al. https://creativecommons.org/licenses/by/4.0/ This content is distributed under the terms of the Creative Commons Attribution 4.0 International license.

10.1128/mSystems.01040-21.6 TABLE S2 Mantel tests of environmental variables against the βNTI of microbial rare and abundant communities. Download Table S2, DOCX file, 0.02 MB. Copyright © 2021 Xu et al. 2021 Xu et al. https://creativecommons.org/licenses/by/4.0/ This content is distributed under the terms of the Creative Commons Attribution 4.0 International license.

### Rare taxa play important roles in bacterial, fungal, and their co-occurrence networks.

**FIG 5:**
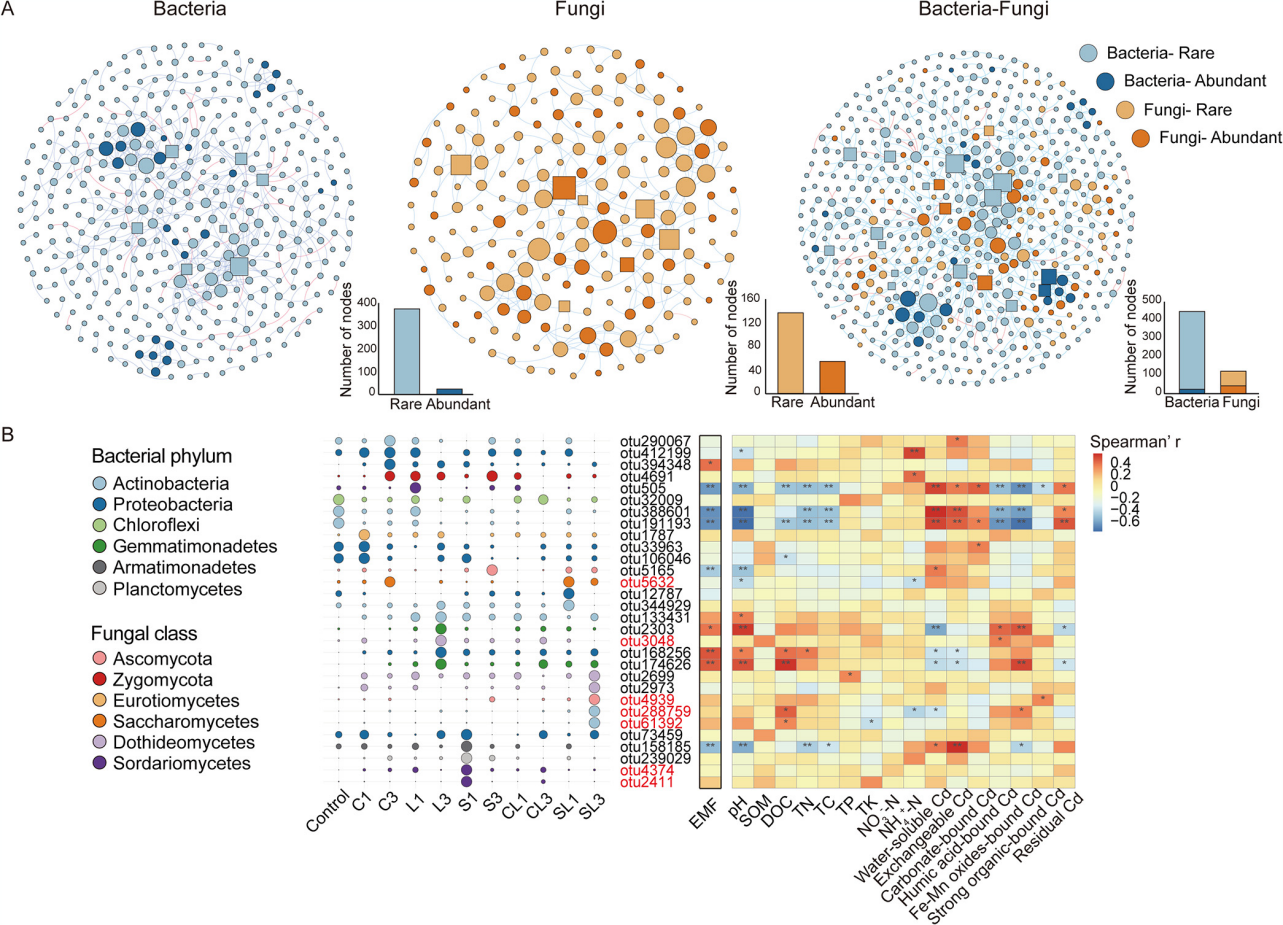
Keystone OTUs and their relationship with environmental variables and ecosystem multifunctionality. (A) Keystone OTUs were identified from bacterial, fungal, and bacterial-fungal cooccurrence networks. Light and dark blue nodes represent rare and abundant bacterial OTUs, while light and dark orange nodes represent rare and abundant fungal OTUs, respectively. Node size is proportional to the number of connections (degree), and the keystone OTUs are represented by square nodes. The blue edges indicate positive interactions between two nodes, and red edges indicate negative interactions. (B) Bubble plot showing the relative abundance (depicted by size) and taxonomy (depicted by color) of the keystone OTUs in each treatment. The names of rare OTUs are marked in black, while the abundant OTUs are red. Spearman correlations between keystone OTUs and ecosystem multifunctionality (EMF) and edaphic factors are shown in the heatmap. The colors in heatmap represent Spearman’s correlation coefficient (*r*), with red being more positive and blue being more negative. Significant correlations were labeled as follows: **, *P* < 0.01; *, *P* < 0.05. Treatments include single application of biochar (C1), CaCO_3_ (L1), straw (S1), CaCO_3_ together with biochar (CL1), and CaCO_3_ together with straw (SL1) and triple application of biochar (C3), CaCO_3_ (L3), straw (S3), CaCO_3_ together with biochar (CL3), and CaCO_3_ together with straw (SL3).

In the bacterial network, rare and abundant OTUs accounted for 93.97% and 6.03% of total nodes, respectively (Fig. 5A). The 7 identified keystone OTUs in the bacterial network were rare OTUs (see Table S3 in the supplemental material). In the fungal network, the proportion of rare OTUs accounted for 71.6% of total nodes with 4 rare and 2 abundant OTUs as keystones (Fig. 5A; see also Table S3). The co-occurrence network of bacterial and fungal communities contained a total of 447 bacterial and 122 fungal nodes (Fig. 5A), among which 5.4% of bacterial and 34.4% of fungal nodes were abundant OTUs. Among 20 keystone OTUs in the bacterial and fungal co-occurrence network, there were 16 bacterial and 4 fungal nodes with an abundant/rare nodes ratio of 1/6 and 3/1, respectively.

10.1128/mSystems.01040-21.7 TABLE S3 Information on keystone OTUs identified from networks. Download Table S3, DOCX file, 0.02 MB. Copyright © 2021 Xu et al. 2021 Xu et al. https://creativecommons.org/licenses/by/4.0/ This content is distributed under the terms of the Creative Commons Attribution 4.0 International license.

Importantly, the types of soil amendment and application frequency altered the distribution of keystone species (Fig. 5B). For instance, triple application of biochar yielded a shift of 3 bacterial keystone species (from otu32009, otu388601, and otu191193 in control to otu290067, otu412199, and otu394348 in triple application of biochar treatment) and an enrichment of a fungal keystone OTU (otu4691). As such, different keystone OTUs were enriched in the single application of straw, single and triple applications of CaCO_3_ with straw, and triple application of CaCO_3_ treatments. Spearman correlation analysis identified 9 keystone OTUs, which were significantly correlated with ecosystem multifunctionality (*P* < 0.05). Keystone OTUs, such as otu394348 in triple application of biochar, otu2303, otu168256, and otu174626 in triple application of CaCO_3_ showed significantly positive correlations with ecosystem multifunctionality. These keystone OTUs may play crucial roles in enhancing ecosystem multifunctionality. Other keystone OTUs, including otu388601 and otu191193 in control, otu505 in single application of CaCO_3_, otu158185 in single application of straw, and otu5165 in triple application of straw, were negatively correlated with ecosystem multifunctionality, suggesting their nonessential roles in improving soil ecosystem multifunctionality.

## DISCUSSION

### Distinct responses of microbial rare and abundant communities to soil amendments.

Understanding the taxonomic and functional changes of rare and abundant communities in response to soil amendments is of great importance for disentangling microbial processes during *in situ* chemical stabilization. Consistent with previous studies (24, 33), the α-diversity of both rare bacterial and fungal communities was obviously higher than abundant communities (see Table S1 in the supplemental material). However, the application of various amendments did not affect α-diversity but markedly altered the community structure of both rare and abundant taxa (Fig. 3). The greater variations in community similarity of rare bacterial taxa between different treatments confirmed our hypothesis that the rare bacterial community was more sensitive to soil amendments than the abundant community (Fig. 3). This result is in line with previous studies showing greater variations in β-diversity of rare bacterial community than those of the abundant community under environmental disturbances (34, 35). The sensitivity of the rare bacterial community could be explained by their narrow environmental breadths to environmental changes (36). In this study, soil pH and Cd speciation exerted greater impacts on the rare bacterial community than the abundant community (see Table S2 in the supplemental material). Further, the environmental breadths of the rare bacterial community were narrower to soil pH and Cd speciation (see Fig. S2 in the supplemental material). The critical roles of soil pH in regulating microbial community have been emphasized in many previous studies (37, 38). During the stabilization process, changes in soil pH are highly related to Cd availability in soil, and the latter has also been reported to affect soil microorganisms (39). However, our observations demonstrated that regulations of soil pH and Cd speciation on rare bacterial taxa were stronger than the abundant taxa.

In contrast to bacteria, the impacts of soil amendments on rare and abundant fungal communities were comparable. Triple application of CaCO_3_ together with biochar yielded the greatest variations in rare and abundant fungal communities, which could be also due to the changes in soil pH and Cd speciation. This explanation was supported by a Monte Carlo permutation test between edaphic factors and fungal communities, showing that abundant fungal community was more affected by soil pH and Cd speciation (Table 1). Importantly, a broader environmental breadth of rare fungal taxa to labile Cd fractions suggested that rare fungal taxa were more resistant to Cd stress and could act as a seed bank to sustain ecological functions in Cd-contaminated soils (15). A similar result has been reported in a previous work documenting that rare fungal taxa are more stable than abundant taxa under different fertilization practices (40). In addition to soil pH and Cd speciation, the abundant fungal community was also more sensitive to DOC (Table 1; Fig. S2). It is reasonable since many fungi prefer soil rich in nutrients and organic matter (41).

### Environmental filtering structured the assembly of rare bacterial community.

Quantifying the relative contributions of deterministic and stochastic processes to microbial community assembly is a key issue to understand forces structuring community composition (42). In this study, we found that deterministic assembly was dominant in the rare bacterial community, while stochastic processes primarily governed the abundant bacterial community (Fig. 4C). Similar observations have been documented in agricultural fields (43) and coastal wetlands (31). The distinct assembly processes between rare and abundant bacterial communities could be due to discrepancies in response and niche breadth to environmental disturbances. It is possible that the rare and abundant taxa occupy distinct ecological niches, which determine their different responses to environmental disturbances (35). Rare bacterial taxa are more likely to be eliminated by environmental filtering due to their narrow niche breadth, while the abundant taxa occupying a broad niche breadth are more resistant to environmental changes (44). Therefore, a narrower niche breadth of rare bacterial community to soil pH and Cd speciation may explain our observation that variable selections govern the assembly of the rare bacterial community (Table S2; Fig. S2). Despite increasing knowledge on the importance of soil pH and organic matter in bacterial community assembly processes (38, 45), our study highlighted that the assembly of the rare bacterial community is more affected by soil pH and Cd speciation, while SOM is crucial for abundant and rare bacterial community assembly processes. Moreover, the significantly lower SES.MNTD values of the rare community indicated a closer phylogenetic clustering by environmental filtering than the abundant community (see Fig. S1A in the supplemental material; *P* < 0.05). Taken together, application of soil amendments altered soil pH, Cd speciation, and organic matter and could further influence community assembly of rare and abundant bacterial taxa.

In line with previous studies showing that the fungal community demonstrates a stronger dispersal limitation than the bacterial community (46, 47), here, we found that the assembly of both abundant and rare fungal communities was dominated by dispersal limitation (Fig. 4D). This is because fungi are more likely to be limited in long‐distance dispersal compared to the smaller‐sized bacteria, as body size of organisms influences their dispersal ability and spatial aggregation (46). However, our result was in contrast to a previous study showing that assembly of rare fungal community was dominated by deterministic process in the agricultural ecosystem (43), possibly due to the differences in habitats and geography.

### Relative importance of rare and abundant microbial taxa in ecosystem multifunctionality.

Restoration of soil ecological function is of importance when assessing the efficiency of *in situ* stabilization strategies (48). In the present study, the evidence from the field trial revealed that repeated application of soil amendments (such as CaCO_3_ and mixture of CaCO_3_ with biochar/straw) promoted the recovery of soil ecosystem multifunctionality (Fig. 1A). Spearman correlation showed that ecosystem multifunctionality had a positive correlation with soil pH and strongly negative correlations with labile Cd fractions. These findings suggested that alleviation of soil acidification and Cd toxicity by soil amendments might contribute to the enhanced ecosystem multifunctionality (Fig. 1B). Considering distinct responses of microbial rare and abundant communities to soil pH and Cd toxicity, we further investigated their relative contributions to ecosystem multifunctionality. Compared to the abundant bacterial community, the rare bacterial community showed a stronger correlation with ecosystem multifunctionality (Fig. 1C). Likewise, a high proportion of rare bacterial keystone species in network analysis further implied the importance of rare taxa (Fig. 5; see also Table S3 in the supplemental material). Meanwhile, we found that both rare and abundant fungal communities were crucial to maintain ecosystem multifunctionality. It is reasonable because fungal species are normally more resistant to heavy metal pollution and play important roles in regulating the ecological functions of contaminated soils (49).

In contrast to previous studies showing that ecosystem multifunctionality is highly related to soil microbial diversity (17, 50), we found that the enhanced ecosystem multifunctionality by soil amendments was not assigned to changes in microbial diversity (Spearman correlation, *P* < 0.05) but due to successions of certain key microbial species. As shown in the distribution of keystone species in different treatments, applications of soil amendments triggered recovery of specific keystone species (Fig. 5B). For instance, triple application of CaCO_3_ induced enrichment of three rare keystone OTUs, including otu2303 and otu174626 belonging to *Gemmatimonadaceae* and otu168256 belonging to *Haliangiaceae*, which were positively correlated with ecosystem multifunctionality. Enrichment of members of *Gemmatimonadaceae* in soil amended with limestone (primarily CaCO_3_) has been reported (51), which are vital species contributing nitrogen cycling and soil respiration in the soil ecosystem (52). Further, the abundances of these three keystone OTUs showed significantly positive correlations with soil pH but a negative correlation with labile Cd fractions. Together, these results suggest that triple application of CaCO_3_ altered soil pH and labile Cd and thereby triggered enrichment of keystone OTUs, which were related to ecosystem multifunctionality. In contrast to CaCO_3_ treatment, application of straw decreased Cd availability via ligand exchange of organic matter rather than changing soil pH. Consequently, an enrichment of otu5165 (*Ascomycota*) was observed in triple application of straw treatment. Members in *Ascomycota* are well known for their ability to degrade lignin and plant residues (53).

In conclusion, this study demonstrated the distinct responses of rare and abundant microbial communities to soil amendments and their relative contributions to ecosystem multifunctionality. Rare bacterial community exhibited greater sensitivity to soil amendments than the abundant community, while the impacts of soil amendments on rare and abundant fungal communities were similar. Soil amendments induced changes in soil pH and Cd speciation and, thereby, influenced the assembly of the rare bacterial community but had limited impacts on the assembly of the abundant bacterial and fungal communities. Furthermore, recovery of specific keystone species by soil amendments may play crucial roles in the restoration of ecosystem multifunctionality in Cd-contaminated soil.

## MATERIALS AND METHODS

### Site description and soil sampling.

A 3-year field experiment for *in situ* stabilization of Cd-contaminated soil was conducted in abandoned agricultural land at the Wangci Village of Daye, Hubei, China (30°03′ N, 114°48′ E). The experimental field is close to a historical mining area and exposed to Cd pollution from mining and smelting operations for centuries (54). Soil in the field is an Alfisol with a silty clay loam texture (82). The total contents of heavy metals in the soil (0 to 20 cm) are as follows: 2.84 mg kg^−1^ Cd, 72.88 mg kg^−1^ copper, 94.62 mg kg^−1^ zinc, 31.48 mg kg^−1^ arsenic, 15.36 mg kg^−1^ cobalt, 51.84 mg kg^−1^ chromium, 27.01 mg kg^−1^ nickel, and 68.65 mg kg^−1^ lead. Prior to the experiment, a full plow tillage was performed at a 20-cm depth after removing herbaceous vegetation grown on the field. A total of 48 plots (10 m^2^ each) with 0.3-m isolation ridges between plots were initially established for Cd stabilization by chemical agents and biological immobilization (see Fig. S4 in the supplemental material). To ensure homogeneous mixture of amendments with soil, a depth of 20 cm soil in plots was dug up and mixed evenly with amendments by harrowing repeatedly. In February 2015, all plots received amendments and were kept for 2 months to ensure that all treatments have achieved a stable condition. To evaluate the effects of Cd stabilization by soil amendments, lettuces (Lactuca sativa L.) were annually sowed in April and harvested in July according to the local growing season. The yield of lettuce harvested in 2017 was used to calculate ecosystem multifunctionality.

10.1128/mSystems.01040-21.4 FIG S4 The scheme of field experiment (A) and the amounts of agents applied in each treatment (B). The plots with a slash in panel A are biological immobilizing treatments that were not included in this study. The timeline shows the key time points for the application of amendments, sowing and harvest of lettuce, and soil sampling. Download FIG S4, TIF file, 2.1 MB. Copyright © 2021 Xu et al. 2021 Xu et al. https://creativecommons.org/licenses/by/4.0/ This content is distributed under the terms of the Creative Commons Attribution 4.0 International license.

To evaluate the efficiency of different chemical agents, we only included chemical-stabilizing treatments and the control treatment in this study (*n* = 33, 11 treatments × 3 replicates). Specifically, the chemical stabilizing treatments were composed of (i) single application of biochar (C1), CaCO_3_ (L1), rice straw (S1), mixture of biochar and CaCO_3_ (CL1), or mixture of rice straw and CaCO_3_ (SL1) in February 2015; and (ii) triple application of above-mentioned agents yearly from 2015 to 2017 (C3, L3, S3, CL3, SL3). All treatments with 3 replicates were randomly distributed in separated plots as illustrated in the scheme of field experiment (see Fig. S4). The amounts of chemical agents were set according to our previous studies on the stabilization of Cd-contaminated agriculture soil (55, 56).

In October 2017, five soil cores (12 cm in depth and 7 cm in diameter) were collected from each plot and pooled together into a sterile ziplock bag. All samples were placed on ice and immediately transported to the laboratory. After removing visible stones, plant residues, and fine roots, soil samples were sieved (2 mm) and mixed homogeneously. The sieved soil samples were divided into two subsamples: one was air-dried and stored at room temperature for soil physicochemical analysis, and the other was freeze-dried and stored at −80°C for DNA extraction and enzyme assay.

### Analysis of soil properties.

Soil pH was measured by a pH meter (PHS-3E; INESA, China) with a soil-water ratio of 2.5:1 (wt/vol). Total carbon (TC) and total nitrogen (TN) contents were determined by a Vario Max element analyzer (Elementar, Germany). Soil organic matter (SOM) content was measured using the potassium dichromate oxidation method (57). Dissolved organic carbon (DOC) was extracted at a soil/water ratio of 1:5 (wt/vol) and measured by a total organic carbon analyzer (multi N/C 2100; Jena, Germany). Inorganic N (NH_4_^+^-N and NO_3_^−^-N) was extracted using 2.0 mol liter^−1^ KCl and determined by a continuous flow analyzer (AA3; SEAL, Germany). Total phosphorus (TP) was determined colorimetrically by the molybdenum blue method after soil digestion using H_2_SO_4_ and HClO_4_ (58). Total potassium (TK) was determined by a flame photometer (M410; Sherwood, England).

The mixture of HF-HClO_4_-HNO_3_ was used to digest soil for the determination of total Cd content (57). Cd speciation was determined using the sequential extraction procedure reported by Tessier with a modification (55, 59). Briefly, the selected extracts were obtained by shaking 2.0 g of soil samples with the following reagents, separately: 20 ml double-distilled water (ddH_2_O) (pH 7.0) for the water-soluble Cd (WS-Cd); 20 ml of 1 M MgCl_2_ (pH 7.0) for the exchangeable Cd (E-Cd); 20 ml of 1 M NaOAc (pH 5.0) for the carbonate-bound Cd (CA-Cd); 40 ml of 1 M Na_4_P_2_O_7_·10H_2_O (pH 10.0) for the humic acid-bound Cd (HS-Cd); 40 ml of 0.25 M NH_2_OH-HCl for the iron-manganese oxide-bound Cd (Fe-MnOx-Cd); 40 ml of the mixtures of 0.02 M HNO_3_, 30% H_2_O_2_, and 3.2 M NH_4_OAc-HNO_3_ for the strong organic-bound Cd (SO-Cd); and 20 ml HNO_3_-HCl-HClO_4_ for the residual Cd (RES-Cd). The concentration of Cd in each fraction was measured using an atomic absorption spectrophotometer (AAS) (AA240FS; Varian, Australia). Details on changes of soil physicochemical properties and Cd speciation in different treatments have been described previously (60). The main changes included a significant increase of soil pH in CL1, L3, CL3, and SL3 treatments compared to that of the control (*P* < 0.05), a notable decrease of water-soluble Cd observed in CL1, SL1, L3, and CL3 treatments (*P* < 0.05), and a significant decline of carbonate-bound Cd in all treatments (*P* < 0.05).

### Quantification of ecosystem multifunctionality.

Ecosystem multifunctionality is defined as the ability of an ecosystem to provide multiple functions and services simultaneously (11). To better reflect the influence of soil amendments on multiple soil processes, 18 ecosystem functions related to nutrient cycling and organic matter turnover were assessed for quantification of the ecosystem multifunctionality using the averaging approach (61). All ecosystem functions were grouped into four categories (11, 17), including (i) plant production—lettuce yield (lettuce harvested in July 2017); (ii) soil conditions—soil pH; (iii) nutrient cycling—TC, TN, ratio of C/N, potential ammonia oxidation (PAO), nitrogen-cycle enzyme (urease), phosphorus-cycle enzyme (phosphatase), functional gene abundance (nitrification genes, denitrification genes, phosphorus cycling genes, and sulfur cycling genes); and (iv) turnover of organic matter—soil basal respiration (SBR), carbon-cycle enzyme (β-glucosidase, β-d-cellulosidase, *N*-acetyl-β-glucosaminidase), and function gene abundance (carbon-fixation and carbon-degradation genes). The ecosystem functions were normalized with Z-score transformation by SPSS v20.0 (SPSS IBM Corp) and averaged to obtain the ecosystem multifunctionality index (EMF) (61).

SBR was determined by gas chromatography (GC-7890 A; Agilent, USA) after incubation of fresh soil (equivalent to 10 g dry mass) in closed 100-cm^3^ soil jars at 25°C. PAO was determined using the chlorate inhibition method (62). The activities of soil extracellular enzymes, including β-glucosidase, β-d-cellulosidase, *N*-acetyl-β-glucosaminidase, and phosphatase were measured fluorometrically by a microplate reader (Spark; Tecan, Switzerland) using 4-methylumbelliferone (MUB)-linked substrates (63). Soil urease activity was measured using the indophenol colorimetry with urea as the substrate (64). Detailed methods on PAO and enzyme activities were described in Text S1 in the supplemental material. The abundances of functional genes involved in carbon fixation, carbon degradation, nitrification, denitrification, phosphorus, and sulfur cycling were measured by GeoChip5.0 (60). The values of 18 ecosystem functions and ecosystem multifunctionality were present in Table S4 in the supplemental material.

10.1128/mSystems.01040-21.8 TABLE S4 Summary of 18 ecosystem functions and ecosystem multifunctionality. Download Table S4, XLSX file, 0.02 MB. Copyright © 2021 Xu et al. 2021 Xu et al. https://creativecommons.org/licenses/by/4.0/ This content is distributed under the terms of the Creative Commons Attribution 4.0 International license.

10.1128/mSystems.01040-21.9 TEXT S1 Determination of potential ammonia oxidation (PAO) and enzyme activities. Download Text S1, DOCX file, 0.02 MB. Copyright © 2021 Xu et al. 2021 Xu et al. https://creativecommons.org/licenses/by/4.0/ This content is distributed under the terms of the Creative Commons Attribution 4.0 International license.

### Soil DNA extraction and high-throughput sequencing.

Soil DNA was extracted from 0.5 g soil using the phenol-chloroform method with the FastPrep system (FastPrep-24; MP, USA) (65). Humic acid was removed by DNA-EZ reagents M Humic acid-Be-Gone B (Sangon Biotech, China). DNA concentration and quality were measured by a NanoDrop spectrophotometer (NanoDrop Technologies, USA). PCR amplifications of bacterial 16S rRNA and fungal internal transcribed spacer (ITS) genes were performed using the primer pairs of 338F/806R (66) and ITS5-1737F/ITS2-2043R (67), respectively. Paired-end sequencing was performed by the Illumina MiSeq PE250 platform (Shanghai Personal Biotechnology Co., Ltd, Shanghai, China). Paired-end reads were assembled using FLASH (68). Quality control and sequence analysis were conducted on the QIIME (v1.8.0) pipeline (69). The criteria for quality control were set as follows: (i) minimum length of 150 bp, (ii) no ambiguous bases, (iii) 5′ end primer mismatch base number < 1, and (iv) the minimum mononucleotide repeats of 8 bp. After removing chimera by UCHIME (70), operational taxonomic units (OTUs) of bacterial and fungal sequences were identified using the UCLUST algorithm with a similarity of 97% (71). Taxonomic classification was conducted based on the Greengenes database 13.8 for bacteria (72) and UNITE database 5.0 for fungi (73). Sequences were rarefied to the minimum sequencing depth at 18,203 for bacteria and 35,883 for fungi.

### Definition of rare and abundant taxa.

To assess the responses of rare and abundant communities to stabilizing treatments, all OTUs were defined and classified into the following 6 categories according to the criteria used in recent studies (25, 74): (i) always abundant taxa (AAT)—OTU with a relative abundance of ≥1% in all samples; (ii) conditionally abundant taxa (CAT)—OTU with a relative abundance of ≥1% in some samples and never <0.01%; (iii) always rare taxa (ART)—OTU with a relative abundance of <0.01% in all samples; (iv) conditionally rare taxa (CRT)—OTU with a relative abundance of <1% in all samples and <0.01% in some samples; (v) moderate taxa (MT)—OTU with a relative abundance between 0.01% and 1% in all samples; and (vi) conditionally rare and abundant taxa (CRAT)—OTU with a relative abundance ranging from rare (<0.01%) to abundant (≥1%). We combined AAT, CAT, and CRAT as abundant taxa and combined ART and CAT as rare taxa for further analyses according to previous studies (74).

### Statistical analysis.

The α-diversity indices of rare and abundant microbial communities were calculated using the “vegan” package in R (version 3.6.3). One-way analysis of variance (ANOVA) with a Student-Newman-Keuls test was performed to test the significance of the differences in microbial α-diversity using the SPSS v20.0. The Bray-Curtis dissimilarity was calculated for rare and abundant communities of bacteria and fungi using the vegdist function in the “vegan” package, with “1-dissimilarity” being used to calculate community similarity (75). The fold change of ecosystem multifunctionality in treatments relative to the control was calculated (*n* = 3). Mantel correlation was employed to measure the relationship between the Bray-Curtis dissimilarity of each community and the Euclidean distance of ecosystem multifunctionalities (76). To identify microbial taxa with significant changes in abundance between treatments and control, differential abundance analyses were carried out using the “DESeq2” package (77). A Monte Carlo permutation test was used to evaluate correlations between edaphic factors and dissimilarity of rare/abundant community for bacteria and fungi using the “vegan” package (permutations = 999; *P* < 0.05). Threshold indicator taxa analysis was used to calculate the threshold values of rare and abundant taxa in response to each environmental variable using the “TITAN2” package (78). The standardized effect size measure of the mean nearest taxon distance (SES.MNTD) was calculated to evaluate the phylogenetic clustering of abundant and rare taxa by the “picante” package (permutations = 999) (79), and the Student’s *t* test was used to compare the means between the rare and abundant groups (*n* = 66). Network analysis was performed to study connections within and between bacterial and fungal taxa using the molecular ecological network analyses (MENA) pipeline with default settings (80) and visualized by Gephi 0.9.2. The keystone OTUs in each network were determined based on the within-module connectivity (Zi) and among-module connectivity (Pi), including network hubs (Zi > 2.5 and Pi > 0.62), module hubs (Zi > 2.5 and Pi < 0.62), and connectors (Zi < 2.5 and Pi > 0.62) (22).

Ecological null modeling was performed to evaluate assembly processes of rare and abundant communities (permutations = 999) (81). β-nearest taxon index (βNTI) quantifies the phylogenetic turnover (phylogenetic β-diversity) and the magnitude and direction of deviation between an observed βMNTD value and the null βMNTD distribution (42). βNTI together with the Raup-Crick metric (RC_bray_) were used to determine contributions from the selective and deterministic ecological processes (81). βNTI was calculated between pairs of communities to estimate the importance of stochasticity and selection using the “picante” package in R. Stochastic or deterministic ecological processes were identified based on the following criteria: (i) βNTI > 2 represents the community assembly driven by variable selection; (ii) βNTI < −2 indicates that homogeneous selection takes a leading role in community assembly; and (iii) |βNTI| < 2 means that the community is mainly assembled by stochastic processes. RC_bray_ was further used to distinguish observed stochastic processes using the “ecodist” package. |βNTI| < 2 and RC_bray_ > 0.95 indicate that the community assembly is dominated by dispersal limitation, |βNTI| < 2 and RC_bray_ < −0.95 indicate that homogenizing dispersal is the dominant assembly process, and |βNTI| < 2 and |RC_bray_| < 0.95 suggest an undominated process (81). After identifying the important edaphic factors correlated with βNTI of abundant and rare communities via Mantel test, regression analysis was used to assess the variation in community assembly processes along the gradients of the derived environmental variables. Permutational multivariate analysis of variance, Monte Carlo permutation test, and Mantel test were conducted using the “vegan” package in R.

### Data availability.

The data sets generated and analyzed during the current study are available in the NCBI SRA database (www.ncbi.nlm.nih.gov/sra) under accession numbers PRJNA601828 for bacteria and PRJNA718172 for fungi.
