# Supplementary figures and images for: Distinct Responses of Rare and Abundant Microbial Taxa to In Situ Chemical Stabilization of Cadmium-Contaminated Soil
Source: mSystems. 2021 Oct 12;6(5):e01040-21. doi: 10.1128/mSystems.01040-21 (PMC8510535; doi:10.1128/mSystems.01040-21)

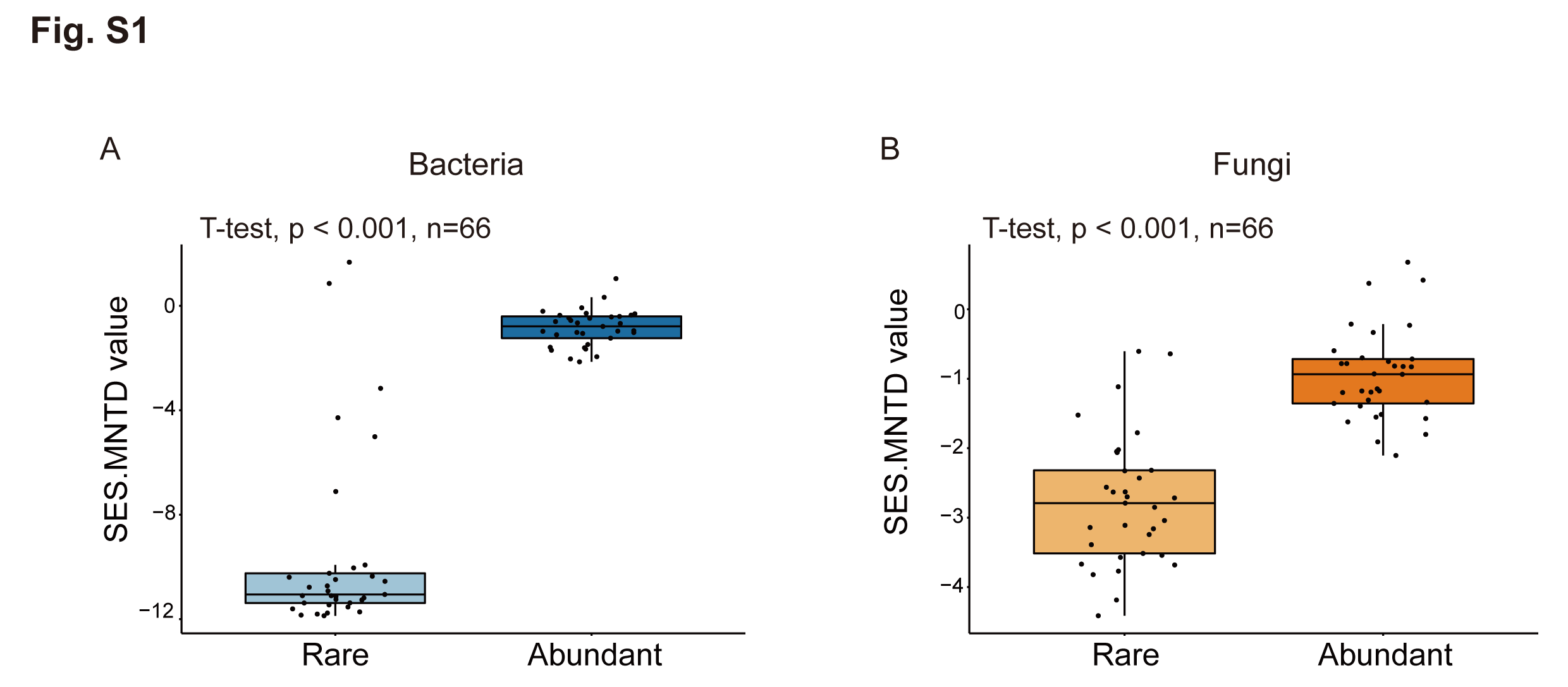

Supplement: FIG S1 [file msystems.01040-21-sf001.tif]

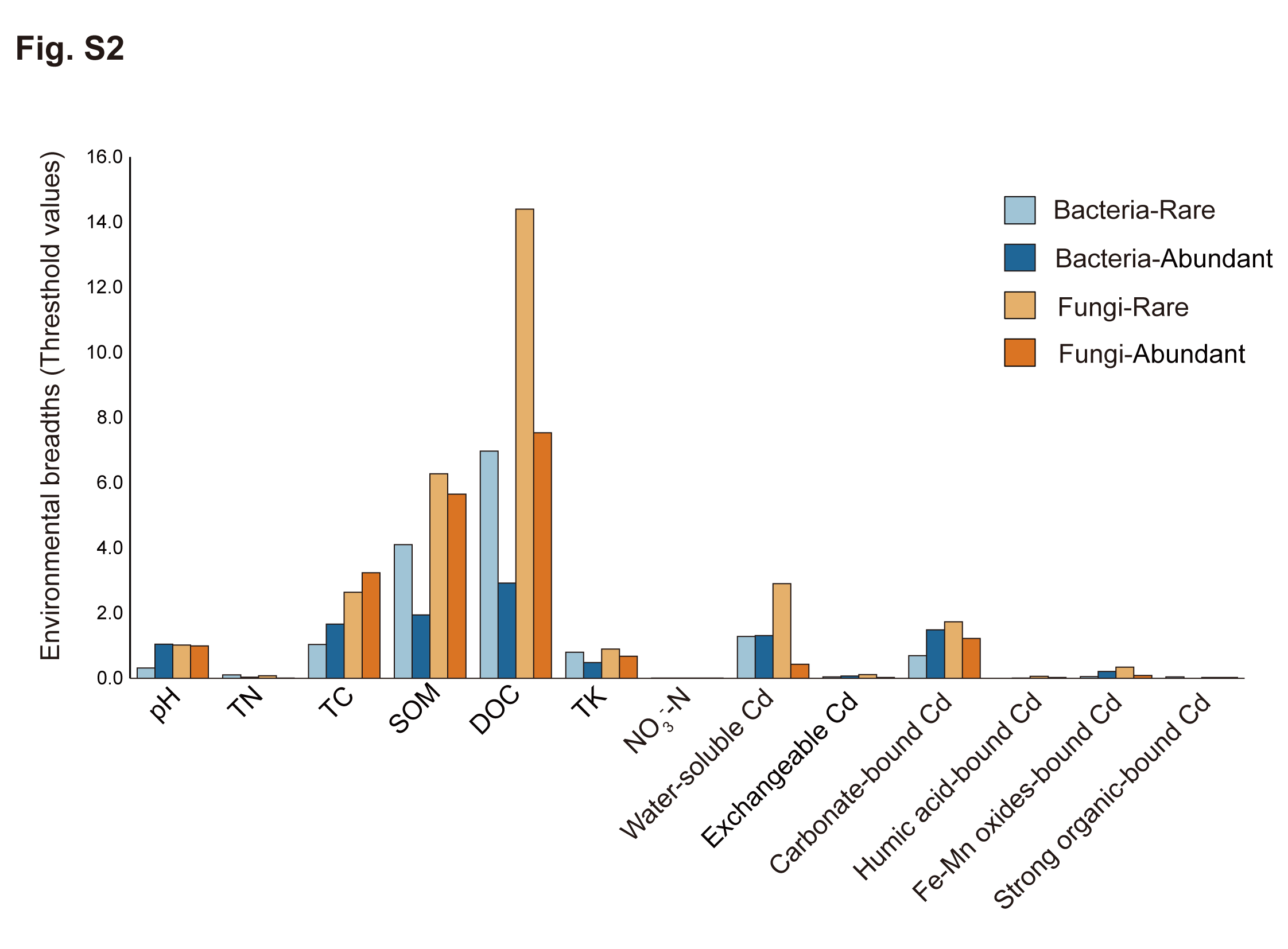

Supplement: FIG S2 [file msystems.01040-21-sf002.tif]

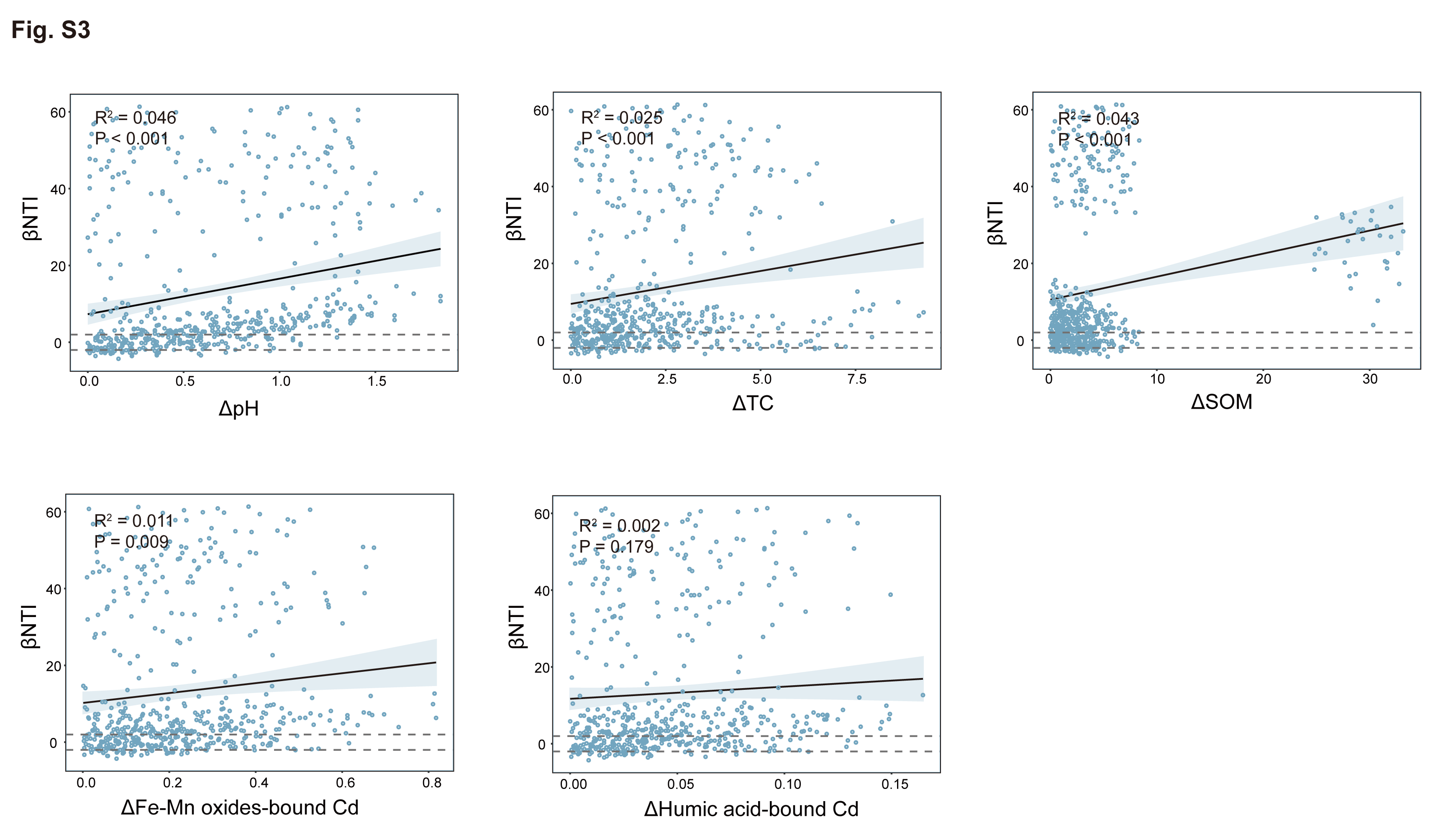

Supplement: FIG S3 [file msystems.01040-21-sf003.tif]

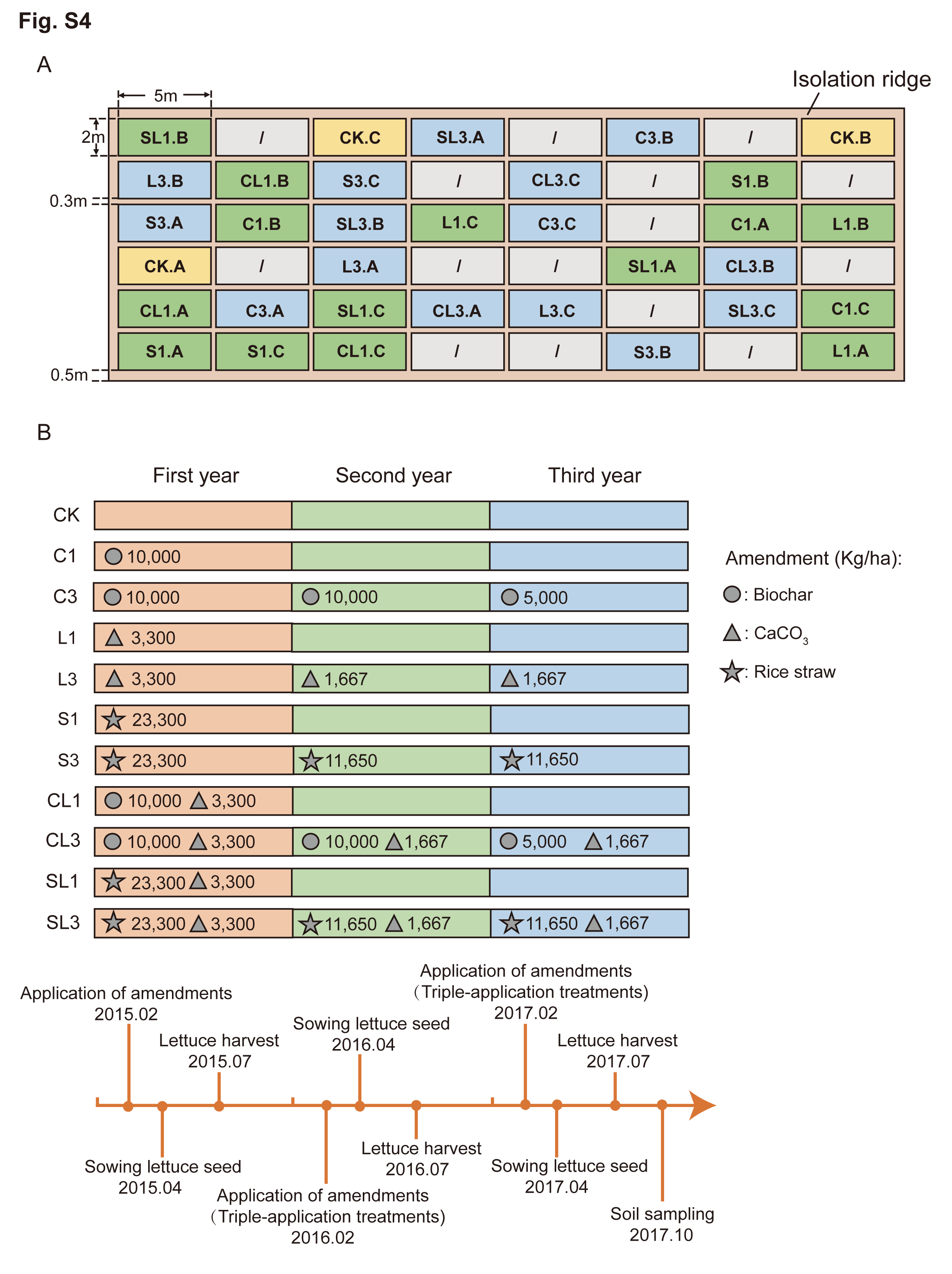

Supplement: FIG S4 [file msystems.01040-21-sf004.tif]
