# Supplementary material for: Distinct Responses of Rare and Abundant Microbial Taxa to In Situ Chemical Stabilization of Cadmium-Contaminated Soil
Source: mSystems. 2021 Oct 12;6(5):e01040-21. doi: 10.1128/mSystems.01040-21 (PMC8510535; doi:10.1128/mSystems.01040-21)
Supplement: TABLE S1 [file msystems.01040-21-st001.docx]

**Table S1 Summary of** α-**diversity index of microbial rare and abundant communities.**

| Sample | Bacteria | | | | Fungi | | | |
| --- | --- | --- | --- | --- | --- | --- | --- | --- |
|  | Rare | | Abundant | | Rare | | Abundant | |
|  | Richness | Shannon | Richness | Shannon | Richness | Shannon | Richness | Shannon |
| CK | 3023±399.387a | 10.578±0.257a | 27±2.494a | 3.551±0.206a | 403±48.231ab | 7.424±0.219ab | 84±2.055a | 4.178±0.299a |
| C1 | 3014±418.144a | 10.687±0.223a | 30±1.247a | 3.539±0.103a | 368±28.083ab | 7.377±0.138ab | 81±3.742a | 4.825±0.055a |
| C3 | 3229±932.739a | 10.63±0.511a | 31±7.846a | 3.684±0.434a | 494±62.537a | 7.823±0.303a | 84±1.886a | 4.984±0.257a |
| L1 | 4176±141.485a | 11.109±0.077a | 25±0.471a | 3.176±0.176ab | 425±6.236ab | 7.464±0.14ab | 88±6.944a | 4.856±0.059a |
| L3 | 3655±277.802a | 10.92±0.085a | 24±2.494a | 2.796±0.09ab | 385±70.542ab | 7.354±0.142ab | 78±6.848a | 4.458±0.175a |
| S1 | 3120±1045.146a | 10.489±0.726a | 26±3.742a | 2.937±0.514ab | 268±71.9b | 6.732±0.515b | 75±3.3a | 3.95±0.904a |
| S3 | 3905±212.539a | 10.989±0.089a | 24±2.867a | 2.536±0.338b | 473±42.434ab | 7.597±0.193ab | 94±2.449a | 4.694±0.311a |
| CL1 | 4050±140.899a | 11.048±0.066a | 23±4.19a | 2.406±0.173b | 424±37.157ab | 7.486±0.132ab | 87±6.481a | 4.55±0.238a |
| CL3 | 2565±947.517a | 10.154±0.569a | 32±3.3a | 3.675±0.321a | 297±64.819ab | 7.054±0.278ab | 79±10.198a | 4.197±0.319a |
| SL1 | 3756±488.404a | 10.994±0.182a | 26±1.414a | 3.419±0.232a | 397±99.71ab | 7.434±0.549ab | 91±3.399a | 3.714±1.071a |
| SL3 | 2502±1138.564a | 10.061±0.81a | 31±7.04a | 3.539±0.315a | 324±91.23ab | 7.236±0.357ab | 80±12.684a | 4.914±0.262a |

Note: Richness was represented by the number of observed OTUs. Different letters mean significant differences between treatments within columns (P < 0.05). Treatments include single-application of biochar (C1), CaCO_3_ (L1), straw (S1), CaCO_3_ together with biochar (CL1), and CaCO_3_ together with straw (SL1), and triple-application of biochar (C3), CaCO_3_ (L3), straw (S3), CaCO_3_ together with biochar (CL3) and CaCO_3_ together with straw (SL3).
