# Supplementary material for: Distinct Responses of Rare and Abundant Microbial Taxa to In Situ Chemical Stabilization of Cadmium-Contaminated Soil
Source: mSystems. 2021 Oct 12;6(5):e01040-21. doi: 10.1128/mSystems.01040-21 (PMC8510535; doi:10.1128/mSystems.01040-21)
Supplement: TABLE S2 [file msystems.01040-21-st002.docx]

**Table S2 Mantel tests of environmental variables against the βNTI of microbial rare and abundant communities.**

|  | Bacteria | | | | Fungi | | | |
| --- | --- | --- | --- | --- | --- | --- | --- | --- |
|  | Rare | | Abundant | | Rare | | Abundant | |
|  | r | p | r | p | r | p | r | p |
| pH | 0.394 | <0.001 | 0.053 | 0.173 | 0.095 | 0.066 | 0.041 | 0.222 |
| TN | 0.013 | 0.385 | 0.139 | 0.038 | 0.018 | 0.392 | 0.095 | 0.066 |
| TC | 0.225 | 0.020 | 0.286 | 0.002 | 0.183 | 0.039 | 0.025 | 0.379 |
| SOM | 0.211 | 0.019 | 0.260 | 0.002 | 0.041 | 0.315 | -0.003 | 0.514 |
| DOC | 0.008 | 0.434 | -0.009 | 0.512 | 0.100 | 0.193 | -0.125 | 0.924 |
| TP | -0.005 | 0.485 | -0.021 | 0.576 | -0.053 | 0.705 | -0.028 | 0.645 |
| TK | 0.088 | 0.146 | -0.077 | 0.839 | 0.160 | 0.034 | -0.031 | 0.671 |
| NO_3_^-^-N | -0.133 | 0.966 | -0.02 | 0.582 | -0.077 | 0.817 | 0.013 | 0.419 |
| NH_4_^+^-N | 0.042 | 0.296 | -0.004 | 0.491 | 0.153 | 0.041 | 0.036 | 0.298 |
| Water-soluble Cd | 0.154 | 0.059 | 0.125 | 0.083 | 0.063 | 0.233 | -0.006 | 0.534 |
| Exchangeable Cd | 0.047 | 0.273 | -0.043 | 0.673 | 0 | 0.479 | -0.072 | 0.829 |
| Carbonate-bound Cd | -0.108 | 0.813 | -0.066 | 0.719 | 0.066 | 0.284 | 0.027 | 0.389 |
| Humic acid-bound Cd | 0.257 | 0.009 | 0.080 | 0.179 | 0.059 | 0.258 | -0.054 | 0.761 |
| Fe-Mn oxides-bound Cd | 0.176 | 0.038 | 0.088 | 0.148 | 0.076 | 0.197 | -0.106 | 0.929 |
| Strong organic-bound Cd | 0.093 | 0.182 | -0.019 | 0.55 | -0.011 | 0.529 | 0.039 | 0.329 |
| Residual Cd | 0.112 | 0.122 | -0.071 | 0.784 | 0.153 | 0.055 | 0.014 | 0.432 |
