# Supplementary material for: Distinct Responses of Rare and Abundant Microbial Taxa to In Situ Chemical Stabilization of Cadmium-Contaminated Soil
Source: mSystems. 2021 Oct 12;6(5):e01040-21. doi: 10.1128/mSystems.01040-21 (PMC8510535; doi:10.1128/mSystems.01040-21)
Supplement: TABLE S3 [file msystems.01040-21-st003.docx]

**Table S3 Information on keystone OTUs identified from networks.**

| Name | Zi | Pi | R/D | Taxonomy | |
| --- | --- | --- | --- | --- | --- |
| Bacterial network | | | | | |
| Module hubs | | | | | |
| otu32009 | 3.27 | 0.00 | Rare | k_Bacteria\| p_Chloroflexi\| c_KD4-96\| o_unidentified\| f_unidentified\| g_unidentified\| s_unidentified | |
| otu12787 | 3.51 | 0.00 | Rare | k_Bacteria\| p_Proteobacteria\| c_Alphaproteobacteria\| o_Sphingomonadales\| f_Sphingomonadaceae\| g_Sphingomonas\| s_uncultured_Sphingomonadaceae_bacterium | |
| otu106046 | 3.23 | 0.20 | Rare | k_Bacteria\| p_Proteobacteria\| c_Alphaproteobacteria\| o_Rhizobiales\| f_Brucellaceae\| g_Ochrobactrum\| s_Ochrobactrum_sp._CGL1 | |
| otu33963 | 3.22 | 0.22 | Rare | k_Bacteria\| p_Proteobacteria\| c_Alphaproteobacteria\| o_Rhizobiales\| f_Brucellaceae\| g_Ochrobactrum\| s_unidentified | |
| otu73459 | 3.31 | 0.55 | Rare | k_Bacteria\| p_Proteobacteria\| c_Betaproteobacteria\| o_Burkholderiales\| f_Comamonadaceae\| g_uncultured\| s_uncultured_bacterium | |
| otu388601 | 3.45 | 0.18 | Rare | k_Bacteria\| p_Actinobacteria\| c_Actinobacteria\| o_Pseudonocardiales\| f_Pseudonocardiaceae\| g_Crossiella\| s_uncultured_bacterium | |
| Connectors |  |  |  |  | |
| otu174626 | -0.56 | 0.67 | Rare | k_Bacteria\| p_Gemmatimonadetes\| c_Gemmaaatimonadetes\| o_Gemmatimonadales\| f_Gemmatimonadaceae\| g_Gemmatimonas\| s_uncultured_Gemmatimonadetes_bacterium | |
| Fungal network | | | | | |
| Module hubs | | | | | |
| otu2973 | 2.88 | 0.34 | Rare | k_Fungi\| p_Ascomycota\| c_Dothideomycetes\| o_Pleosporales\| f_unidentified\| g_unidentified\| s_Pleosporales sp | |
| otu4374 | 3.24 | 0.15 | Abundant | k_Fungi\| p_Ascomycota\| c_Sordariomycetes\| o_Hypocreales\| f_Incertae sedis\| g_Acremonium\| s_Acremonium curvulum | |
| otu1787 | 2.84 | 0.00 | Rare | k_Fungi\| p_Ascomycota\| c_Eurotiomycetes\| o_Chaetothyriales\| f_unidentified\| g_unidentified\| s_Chaetothyriales sp | |
| Connectors |  |  |  |  | |
| otu2699 | 1.24 | 0.64 | Rare | k_Fungi\| p_Ascomycota\| c_Dothideomycetes\| o_Pleosporales\| f_Phaeosphaeriaceae\| g_unidentified\| s_Phaeosphaeriaceae sp | |
| otu4939 | 0.37 | 0.67 | Abundant | k_Fungi\| p_Ascomycota\| c_unidentified\| o_unidentified\| f_unidentified\| g_unidentified\| s_Ascomycota sp | |
| otu505 | -1.09 | 0.67 | Rare | k_Fungi\| p_Ascomycota\| c_Sordariomycetes\| o_Sordariales\| f_unidentified\| g_unidentified\| s_Sordariales sp | |
| otu4691 | -0.39 | 0.63 | Rare | k_Fungi\| p_Zygomycota\| c_Incertae sedis\| o_Basidiobolales\| f_Basidiobolaceae\| g_Schizangiella\| s_Schizangiella serpentis | |
| Bacteria-fungal network | | | | | |
| Module hubs | | | | | |
| otu191193 | 2.64 | 0.00 | Rare | | k_Bacteria\| p_Actinobacteria\| c_Actinobacteria\| o_Frankiales\| f_Acidothermaceae\| g_Acidothermus\| s_uncultured_bacterium |
| otu61392 | 2.57 | 0.00 | Abundant | | k_Bacteria\| p_Actinobacteria\| c_Actinobacteria\| o_Micrococcales\| f_Micrococcaceae\| g_unidentified\| s_unidentified |
| otu288759 | 2.95 | 0.37 | Abundant | | k_Bacteria\| p_Actinobacteria\| c_Actinobacteria\| o_Micrococcales\| f_Micrococcaceae\| g_Pseudarthrobacter\| s_uncultured_bacterium |
| otu344929 | 2.56 | 0.00 | Rare | | k_Bacteria\| p_Actinobacteria\| c_MB-A2-108\| o_uncultured_bacterium\| f_uncultured_bacterium\| g_uncultured_bacterium\| s_uncultured_bacterium |
| otu158185 | 2.87 | 0.12 | Rare | | k_Bacteria\| p_Armatimonadetes\| c_Chthonomonadetes\| o_Chthonomonadales\| f_Chthonomonadaceae\| g_Chthonomonas\| s_unidentified |
| otu32009 | 3.60 | 0.00 | Rare | | k_Bacteria\| p_Chloroflexi\| c_KD4-96\| o_unidentified\| f_unidentified\| g_unidentified\| s_unidentified |
| otu239029 | 3.39 | 0.10 | Rare | | k_Bacteria\| p_Planctomycetes\| c_Phycisphaerae\| o_Phycisphaerales\| f_Phycisphaeraceae\| g_SM1A02\| s_uncultured_bacterium |
| otu394348 | 2.58 | 0.61 | Rare | | k_Bacteria\| p_Proteobacteria\| c_Alphaproteobacteria\| o_Rhodospirillales\| f_Rhodospirillales_Incertae_Sedis\| g_Candidatus_Alysiosphaera\| s_uncultured_bacterium |
| otu12787 | 2.88 | 0.20 | Rare | | k_Bacteria\| p_Proteobacteria\| c_Alphaproteobacteria\| o_Sphingomonadales\| f_Sphingomonadaceae\| g_Sphingomonas\| s_uncultured_Sphingomonadaceae_bacterium |
| otu73459 | 4.65 | 0.44 | Rare | | k_Bacteria\| p_Proteobacteria\| c_Betaproteobacteria\| o_Burkholderiales\| f_Comamonadaceae\| g_uncultured\| s_uncultured_bacterium |
| otu388601 | 3.23 | 0.34 | Rare | | k_Bacteria\| p_Actinobacteria\| c_Actinobacteria\| o_Pseudonocardiales\| f_Pseudonocardiaceae\| g_Crossiella\| s_uncultured_bacterium |
| otu3048 | 3.13 | 0.00 | Abundant | | k_Fungi\| p_Ascomycota\| c_Dothideomycetes\| o_Pleosporales\| f_Incertae sedis\| g_Phoma\| s_Phoma calidophila |
| otu5165 | 3.06 | 0.24 | Rare | | k_Fungi\| p_Ascomycota\| c_unidentified\| o_unidentified\| f_unidentified\| g_unidentified\| s_Ascomycota sp |
| otu2411 | 3.17 | 0.35 | Abundant | | k_Fungi\| p_Ascomycota\| c_Sordariomycetes\| o_Hypocreales\| f_Incertae sedis\| g_unidentified\| s_Incertae sedis sp |
| otu5632 | 2.88 | 0.58 | Abundant | | k_Fungi\| p_Ascomycota\| c_Saccharomycetes\| o_Saccharomycetales\| f_Incertae sedis\| g_Candida\| s_Candida xylopsoci |
| Connector | | | | | |
| otu133431 | 0.00 | 0.63 | Rare | | k_Bacteria\| p_Actinobacteria\| c_Actinobacteria\| o_Propionibacteriales\| f_Nocardioidaceae\| g_Nocardioides\| s_uncultured_bacterium |
| otu290067 | -0.87 | 0.75 | Rare | | k_Bacteria\| p_Actinobacteria\| c_Acidimicrobiia\| o_Acidimicrobiales\| f_uncultured\| g_uncultured_bacterium\| s_uncultured_bacterium |
| otu2303 | 1.50 | 0.64 | Rare | | k_Bacteria\| p_Gemmatimonadetes\| c_Gemmatimonadetes\| o_Gemmatimonadales\| f_Gemmatimonadaceae\| g_uncultured\| s_uncultured_bacterium |
| otu412199 | -0.54 | 0.67 | Rare | | k_Bacteria\| p_Proteobacteria\| c_Deltaproteobacteria\| o_Myxococcales\| f_uncultured\| g_uncultured_bacterium\| s_uncultured_bacterium |
| otu168256 | -0.67 | 0.67 | Rare | | k_Bacteria\| p_Proteobacteria\| c_Deltaproteobacteria\| o_Myxococcales\| f_Haliangiaceae\| g_Haliangium\| s_unidentified |
