# Supplementary material for: Distinct Responses of Rare and Abundant Microbial Taxa to In Situ Chemical Stabilization of Cadmium-Contaminated Soil
Source: mSystems. 2021 Oct 12;6(5):e01040-21. doi: 10.1128/mSystems.01040-21 (PMC8510535; doi:10.1128/mSystems.01040-21)
Supplement: TEXT S1 [file msystems.01040-21-s0001.docx]

**Supplemental Text S1**

**Determination of potential ammonia oxidation (PAO) and enzyme activities**

PAO rates were determined by the chlorate inhibition method (1). In brief, soil (equivalent to 5g dry mass) was mixed with 50 ml of 2 mM (NH_4_)_2_SO_4_ and 10 mM NaClO_3_. The suspension was shaken at 150 rpm at 25 °C for 72 h. During the incubation, 1 ml aliquots were taken at 0, 8, 24, 30, 48, 60 and 72 h, and centrifuged at 5000 rpm for 5 min. The suspension was filtered through 0.2 mm membrane filters, and used for the measurement of nitrite accumulation. The rates were calculated from the linear increase and taken as PAO. The activities of β-glucosidase, β-D-cellulosidase, N-acetyl-β-glucosaminidase and phosphatase were determined fluorometrically in 96-well microplates using 4-methylumbelliferone (MUB)-linked substrates (2). Briefly, a volume of 50 μL MUB standard (0, 2.5, 5, 10, 25, 50, 100 μM) was prepared and pipetted into corresponding wells of the MUB standard plate. Soil slurries were prepared via blending 1g soil with 125 ml of sodium acetate buffer (50 mM , pH 5.0).

Volume of 200 μL mixture of soil slurries and sodium acetate buffer was pipetted into corresponding wells of the standard plate and sample plate, respectively. Then 50 μL appropriate substrate (200 μM) were pipetted into corresponding wells of the sample plate. All microplates were incubated for 1.5 h at 30°C. Fluorescence was measured by a microplate reader (TECAN, Spark, Switzerland) with 365 nm excitation and 450 nm emission filters. The enzyme activities were expressed as nmol of substrate released per gram of dry soil per hour (nmol g soil^−1^ h^−1^). Soil urease activity was measured by the indophenol colorimetry using urea as a substrate (3). The quantity of ammonium released over 24 h at 37°C was measured colorimetrically at 578 nm. Soil urease activity was expressed as a mg g^-1^ dry sample after 24 h.

**REFERENCES**

1. Ke X, Angel R, Lu Y, Conrad R. 2013. Niche differentiation of ammonia oxidizers and nitrite oxidizers in rice paddy soil. *Environ Microbiol* **15**:2275-92.

2. Bell CW, Fricks BE, Rocca JD, Steinweg JM, McMahon SK, Wallenstein MD. 2013. High-throughput fluorometric measurement of potential soil extracellular enzyme activities. *Jove-J Vis Exp* **81**:50961.

3. Shang LR, Wan LQ, Zhou XX, Li S, Li XL. 2020. Effects of organic fertilizer on soil nutrient status, enzyme activity, and bacterial community diversity in Leymus chinensis steppe in Inner Mongolia, China. *Plos One* **15**.
